# Comparison of Visual Field Progression Rate before and after Cataract Surgery in Patients with Open-Angle and Angle-Closure Glaucoma

**DOI:** 10.1155/2021/7655747

**Published:** 2021-06-22

**Authors:** Wei-Wen Su, Shian-Sen Hsieh, Ming-Hui Sun, Henry Shen-Lih Chen, Yung-Sung Lee, Lan-Yan Yang, Hsiao-Jung Tseng

**Affiliations:** ^1^Department of Ophthalmology, Chang Gung Memorial Hospital, Linkou, Taiwan; ^2^Chang Gung University College of Medicine, Taoyuan, Taiwan; ^3^Department of Internal Medicine, Chang Gung Memorial Hospital, Linkou, Taiwan; ^4^Biostatistics Unit, Clinical Trial Center, Chang Gung Memorial Hospital, Linkou, Taiwan

## Abstract

**Purpose:**

To evaluate Humphrey visual field (VF) progression rate before and after cataract surgery in patients with open-angle glaucoma (OAG) and angle-closure glaucoma (ACG).

**Methods:**

After reviewing all records in our Humphrey VF database from July 1, 2001, to December 31, 2018, eyes of OAG and ACG that had received uncomplicated phacoemulsification with intraocular lens implantation during the period and had been followed up regularly for more than one year with ≥3 reliable VF tests before and after cataract surgery, respectively, were enrolled. The VF progression rate, visual acuity, and intraocular pressure (IOP) before and after cataract surgery were compared.

**Results:**

Seventy-seven eyes (OAG: 51; ACG: 26) from 60 patients were enrolled. The mean preoperative and postoperative follow-up durations were 4.89 ± 2.70 and 5.48 ± 2.30 years in the OAG group and 5.14 ± 3.31 and 5.97 ± 2.35 years in the ACG group. IOP and visual acuity significantly improved postoperatively in both groups. In the OAG group, there was no significant change in the VF progression rate (pre-op: −0.39 ± 0.94 dB/year; post-op: −0.47 ± 0.72 dB/year) (*P*=0.619), whereas, in the ACG group, the rate significantly flattened after surgery from −1.39 ± 1.31 dB/year to −0.34 ± 0.64 dB/year (*P*=0.003).

**Conclusions:**

The VF progression rate did not differ between preoperative and postoperative eyes with OAG, but those with ACG exhibited rapid VF decline preoperatively, which was significantly flattened postoperatively.

## 1. Introduction

Cataract is commonly observed in patients with glaucoma, not only because the prevalence of both diseases increases with age [1, 2] but also because the treatment for glaucoma, whether medical or surgical, increases the rate of cataract development [3, 4]. Cataract causes diffuse loss of sensitivity on automated perimetry in patients with glaucoma, making the detection of progression more challenging. Cataract extraction improves visual acuity as well as the global measures of visual field (VF), such as mean deviation (MD) and VF index (VFI). Previous studies have reported an improvement in MD in glaucomatous eyes after cataract extraction [5–7]; however, most results are based on a comparison of only one or two VF tests preoperatively and postoperatively [5–12]. Few studies have addressed long-term changes in VF progression. With a median follow-up of 6.5 years before cataract surgery and 5.3 years after it, Kim et al. reported that, in primary open-angle glaucoma (POAG), all VF indices indicated accelerated decay postoperatively [13]. By contrast, Lee et al. followed up patients with OAG for a mean of 5.6 years before cataract surgery and 6.0 years after it and found no statistically significant differences in MD or VF decay rates [14].

Cataract extraction is an effective method for reducing intraocular pressure (IOP) in patients with glaucoma, particularly in narrow-angle eyes. Medical burden can also be considerably reduced through adoption of this approach. Moreover, lower IOP aids the stabilization of VF progression. The EAGLE study indicated that, even in patients with angle-closure glaucoma (ACG) without any obvious signs of cataract, clear-lens extraction demonstrated greater efficacy and was more cost effective than laser peripheral iridotomy (LPI) in IOP control. In addition, it was associated with a reduced requirement for medications and subsequent glaucoma surgery [15]. However, the influence of lens extraction on VF progression remains to be elucidated.

In this study, our aim was to compare the progression rate of Humphrey VF before and after cataract surgery in patients with OAG and ACG and evaluate whether cataract extraction reduces the rate of VF decline.

## 2. Materials and Methods

This study was approved by the Chang Gung Medical Foundation Institutional Review Board and was conducted in accordance with the tenets set forth in the Declaration of Helsinki.

### 2.1. VF Tests Screening

**Figure 1:**
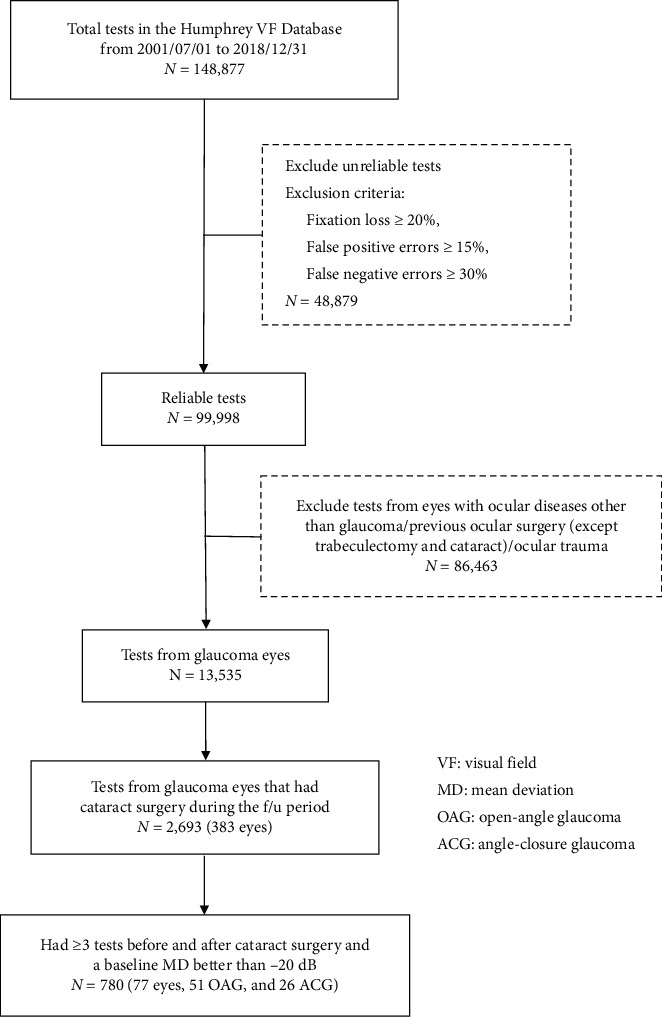
Flowchart showing the inclusion of 77 eyes/780 tests from our Humphrey VF database that had been followed up for more than one year with at least three reliable VF tests before and after cataract surgery, respectively, and a baseline MD better than -20 dB.

We reviewed all VF records in the Humphrey VF database of Chang Gung Memorial Hospital Linkou Branch from July 1, 2001, to December 31, 2018. A total of 148,877 tests were conducted. After excluding unreliable tests, defined as fixation loss ≥20%, false positive errors ≥15%, and false negative errors ≥30%, 99,998 tests were left. Then, we excluded tests from eyes that had ocular diseases other than glaucoma, had previous ocular surgery except trabeculectomy or cataract, or had a history of ocular trauma. The remaining tests were collected for further analysis if the tests were from eyes that had received uncomplicated phacoemulsification with intraocular lens (IOL) implantation during the follow-up period, had been followed up for more than one year with at least three VF tests before and after cataract surgery, respectively, and had a baseline MD better than −20 dB.  Figure 1 shows the flowchart of the inclusion process.

### 2.2. Perimetric Tests

All VF tests were performed using the Humphrey Field Analyzer (Carl Zeiss Meditec Inc., Dublin, CA, USA) with a 30-2 test pattern and the standard size III stimulus Swedish interactive threshold algorithm at a regular interval of approximately every six months. The last VF test before cataract surgery and the first one after the surgery were required to be within one year from cataract surgery.

### 2.3. Visual Field Data Analysis

After removal of the edge points (except the two points closest to the nose across the horizontal midline) and the two points corresponding to the blind spot, raw data pertaining to the retinal sensitivity of the remaining 52 test locations of each VF test were extracted and averaged. The averaged retinal sensitivity (mean sensitivity [MS]) was regressed over time, and the annual rate of change (MS slope [dB/year]) was calculated preoperatively and postoperatively.

### 2.4. Glaucoma Classification

OAG was defined as the presence of an open angle observed through gonioscopy and glaucomatous optic neuropathy (GON) with correlating VF defects. ACG was defined as the presence of an occludable or closed drainage angle (posterior trabecular meshwork not visible at >180° through gonioscopy), elevated IOP, GON, and glaucomatous VF defects. All glaucomatous eyes were treated with various forms of medical or surgical therapy. All patients with ACG received LPI.

### 2.5. Cataract Surgeries

All cataract surgeries were performed by experienced anterior segment surgeons with clear-corneal, small-incisional phacoemulsification. All patients received an acrylic foldable, monofocal IOL implanted in a capsular bag. The age at surgery, cataract morphology, baseline MD, preoperative (obtained from the last record before surgery) and postoperative (records of the third month after surgery) best-corrected visual acuity (BCVA, converted into logarithm of the minimum angle of resolution, logMAR), IOP, vertical cup to disc ratio (CDR), number of medications for glaucoma, follow-up duration, number of VF tests, and MS slope were evaluated.

### 2.6. Statistical Analysis

Statistical analyses were performed using SPSS v23.0 for Windows (SPSS Inc., Chicago, IL, USA). Continuous variables are expressed as the mean ± standard deviation (SD) or median with range in the parentheses. Categorical variables are expressed as count and percentage, as well. Differences between OAG and ACG were compared using independent *t*-test or Mann–Whitney *U* test. Categorical data were compared using the X2 test or fisher's exact test. A general linear model with repeated measurements was used to compare preoperative and postoperative as well as group difference. *P* < 0.05 indicates statistical significance.

## 3. Results

A total of 780 VF tests from 77 eyes (60 patients) were included. Among them, 51 eyes were OAG and 26 eyes were ACG. The OAG patients mainly consisted of POAG and a small number of secondary OAG, including one case of pigmentary glaucoma. Seven eyes in the OAG group (13.7%) and six in the ACG group (23.1%) had a history of trabeculectomy, which was performed years before cataract surgery.  Table 1 presents the demographic and clinical characteristics of study participants.

**Figure 2:**
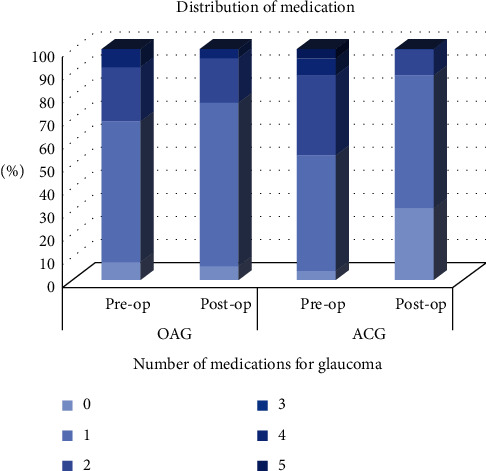
Distribution of medication in OAG and ACG before and after cataract surgery.

The mean surgical age was 58.96 ± 11.64 years in the OAG group and 68.15 ± 13.67 years in the ACG group. Posterior subcapsular (PSC) cataract was presented in 20 (39.2%) of the OAG patients and seven (26.9%) of the ACG patients (*P*=0.279). The baseline MD was −7.09 ± 5.01 in the OAG group and −6.52 ± 5.44 in the ACG group (*P*=0.383). The median (range) follow-up duration before and after cataract surgery was 4.48 (1.09–14.22) and 5.25 (1.49–10.80) years, respectively, in the OAG group and 4.83 (1.01–12.63) and 6.07 (1.10–9.73) years, respectively, in the ACG group. The median (range) of VF tests performed before and after cataract surgery was 4 (3–14) and 4 (3–19), respectively, in the OAG group and 3 (3–8) and 4 (3–8), respectively, in the ACG group. The median (range) preoperative and postoperative number of medications for glaucoma in the OAG group was 1 (0–3) and 1 (0–3), and in the ACG group, it was 1 (0–5) and 1 (0–2), respectively. The number of medications for glaucoma postoperatively was significantly lower in the ACG group than in the OAG group (*P*=0.009; Table 1).  Figure 2 shows the distribution of the number of medications for glaucoma in the OAG and ACG patients before and after cataract surgery.

The MS and variance (presented in SD) of the last VF before cataract surgery and the first VF after cataract surgery are shown in Table 2. There was no significant difference in the MS, but the postoperative variance was larger in both groups.

**Figure 3:**
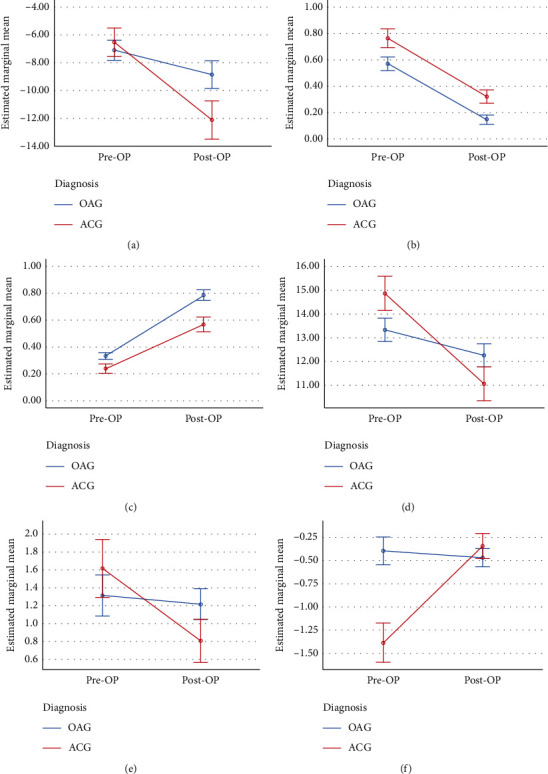
ANOVA group-time interaction effect on (a) MD, (b) logMAR, (c) Snellen VA, (d) IOP, (e) number of medications, and (f) MS slope in OAG and ACG. MD: mean deviation; logMAR: logarithm of the minimum angle of resolution; VA: visual acuity; IOP: intraocular pressure; MS: mean sensitivity; OAG: open-angle glaucoma; ACG: angle-closure glaucoma.

After cataract surgery, the mean logMAR BCVA significantly improved from 0.57 ± 0.32 to 0.18 ± 0.34 in the OAG group and from 0.76 ± 0.41 to 0.30 ± 0.29 in the ACG group. The mean IOP decreased from 13.33 ± 3.37 to 12.26 ± 3.64 in the OAG group and from 14.87 ± 3.81 to 11.12 ± 2.92 in the ACG group (Table 3). The MS slope before and after cataract surgery was −0.39 ± 0.94 dB/year and −0.47 ± 0.72 dB/year, respectively, in the OAG group (*P*=0.619) and −1.39 ± 1.31 dB/year and −0.34 ± 0.64 dB/year, respectively, in the ACG group (*P*=0.003) (Table 3).  Figure 3 shows the ANOVA group-time interaction effect on MD, logMAR, Snellen VA, IOP, number of medications, and MS slope.

## 4. Discussion

In this study, patients with OAG and ACG exhibited improved visual acuity and IOP after cataract surgery. In the OAG group, the preoperative and postoperative MS slopes were similar, whereas, in the ACG group, the MS slope was significantly flattened after cataract surgery. The number of medications for glaucoma also significantly decreased postoperatively in the ACG group.

In the current study, the cataract morphology of the two groups was similar, and the preoperative MD of the two groups did not reach statistical significance. Cataract results in diffuse loss of sensitivity in automated perimetry. Cataract extraction removes generalized depression, thereby improving global VF measures. MD is the average of all differences between measures and their normal values, weighted by variances observed in the general population [16]. Previous studies involving patients with glaucoma who underwent cataract extraction have reported varied changes in MD, ranging from no significant change [17, 18] to significant improvements [5, 6, 8, 19]. In general, cataract extraction should result in a significant improvement in MD and VFI, but the change in pattern standard deviation, an indicator of localized field loss, has varied considerably [5–7].

Most studies have focused on the short-term effects of cataract extraction on visual acuity and VF parameters, and its long-term effects on VF remain to be elucidated. Lee et al. evaluated the effect of cataract surgery on VF decay in patients with OAG. They found no statistically significant difference in MD decay rates postoperatively; furthermore, the decay rate of the fast component, which represented changes from glaucoma, was not affected by cataract surgery [14]. By contrast, Kim et al. reported that, in POAG, all VF indices exhibited accelerated decay after cataract surgery. In their report, the MD slope worsened from −0.18 ± 0.40 dB/year preoperatively to −0.40 ± 0.62 dB/year postoperatively (*P* < 0.001) [13]. Unlike previous studies, we used the MS of the central 52 test locations for regression analyses instead of the MD slope, because eyelid artifacts and lens rim artifacts were common in our patients, and the edge point data had to be removed to eliminate these errors. Herein, we noted no significant changes in MS between immediately before and after cataract surgery in either the OAG or ACG group. However, the variance was significantly higher postoperatively. Theoretically, the observed MS slope should have included age-related decline in VF, which has been reported to be between −0.064 and −0.172 dB/year [20–22]. The MS slope should be slightly larger than the MD slope, although the difference is small, particularly after the exclusion of edge points. De Moraes et al. used the MS slope to evaluate VF progression outcomes in glaucoma subtypes, and they reported that the MS slope was −0.48 ± 0.8 dB/year in patients with POAG and −0.33 ± 0.3 dB/year in those with normal‐tension glaucoma [23]. In this study, the VF decline rate in patients with OAG was comparable to previously reported data.

The VF decline rate observed in our ACG patients was much higher than previously reported by De Moraes et al. and Ballae et al. The former reported an MS slope of −0.39 ± 0.5 dB/year for ACG patients [23], while the latter reported a baseline MD-matched or age-matched MD slope of −0.4 to −0.42 dB/year for patients with phakic primary ACG [24]. In the current study, the preoperative IOP of the ACG eyes was similar to that of the OAG eyes; however, the VF decline rate was three times faster. According to the design of this study, the preoperative IOP was obtained from the last outpatient record before cataract surgery. It is likely that a single, office-hour IOP record might not provide sufficient information for determining the long-term fluctuations in IOP. Herein, all eyes with ACG had received LPI. LPI relieves pupillary block, stabilized the IOP, and may halt the progression of angle closure [25]. Thus, LPI is strongly recommended in eyes with primary angle-closure or primary angle-closure glaucoma. However, after LPI, there was an initial increase in angle width, followed by a gradual narrowing of the angle over time, attributable to lens change [26]. Age-related growth of the lens plays a major role in the pathogenesis of ACG. Lens extraction effectively deepens the anterior chamber and widens the angle [27]. According to the EAGLE study, clear-lens extraction demonstrated greater efficacy in controlling IOP and was more cost effective than conventional LPI [15]. However, the VF decay rate before and after lens extraction was not reported in the EAGLE study. The current study showed that, before cataract surgery, eyes with ACG progressed more rapidly than eyes with OAG, although IOP seemed to be well controlled. After cataract surgery, the VF decline rate in the ACG group significantly flattened, and there was no statistical difference between the pseudophakic ACG and OAG eyes. Cataract extraction not only resulted in better vision and lower IOP but also delayed VF decline in patients with ACG.

This study has a few limitations. Although our patients received regular VF checks every six months, some tests failed to pass the strict reliability requirement and were discarded initially, which reduced the sample size and decreased the statistical power. The retrospective design may have introduced selection or information bias. The effect of cataract morphology on VF test results was not addressed. The extent of preoperative PAS in the ACG group was not analyzed. The ACG group had trabeculectomy more commonly than the OAG group, which was a bias that would decrease the risk of progression. In addition, our results are specific to East Asian eyes and involved a tertiary glaucoma clinic; thus, they may not be generalizable to other populations.

In conclusion, we found that the rate of VF decline did not change after cataract surgery in the eyes with OAG and eyes with ACG exhibited rapid VF decline preoperatively that significantly slowed down postoperatively.

## Figures and Tables

**Table 1:** Demographics and clinical characteristics among 77 eyes with open-angle and angle-closure glaucoma.

	OAG (*n* = 51)	ACG (*n* = 26)	*P*
History of trabeculectomy	7 (13.7%)	6 (23.1%)	0.344

Surgical age	58.96 ± 11.64	68.15 ± 13.67	0.002

Preoperative logMAR	0.57 ± 0.32	0.76 ± 0.41	0.014

Cataract morphology			
NS/CO cataract	48 (94.1%)	26 (100%)	0.083
PSC cataract	20 (39.2%)	7 (26.9%)	0.279

Preoperative IOP	13.33 ± 3.37	14.87 ± 3.81	0.175

No. of medications for glaucoma			0.604
Before surgery	1 (0–3)	1 (0–5)	0.181
After surgery	1 (0–3)	1 (0–2)	0.009

Preoperative vertical CDR	0.77 ± 0.20	0.79 ± 0.16	0.720

Baseline MD	−7.09 ± 5.01	−6.52 ± 5.44	0.383

Total assessment duration (years)	9.95 (4.15–16.96)	11.30 (4.17–16.56)	0.166
Before surgery	4.48 (1.09–14.22)	4.83 (1.01–12.63)	0.927
After surgery	5.25 (1.49–10.80)	6.07 (1.10–9.73)	0.380

Total no. of VF tests			0.897
6–10 tests	38 (74.51%)	21 (80.77%)	
11–15 tests	11 (21.57%)	5 (19.23%)	
15+ tests	2 (3.92%)	0 (0.00%)	
Before surgery	4 (3–14)	3 (3–8)	0.054
After surgery	4 (3–19)	4 (3–8)	0.798

Durations, VF tests, and numbers of medications are presented as median and range due to data skewness. Mann–Whitney *U* test (continuous) or Fisher's exact test (categorical). OAG: open-angle glaucoma; ACG: angle-closure glaucoma; logMAR: logarithm of the minimum angle of resolution; NS/CO: nuclear sclerosis/cortical opacity; PSC: posterior subcapsular; IOP: intraocular pressure; CDR: cup to disc ratio; MD: mean deviation; VF: visual field.

**Table 2:** Change in mean sensitivity and variance of VF before and after cataract surgery in patients with OAG and ACG.

	Last VF preoperatively	First VF postoperatively	*P*
OAG			
Mean sensitivity (dB)	21.14 ± 6.31	21.70 ± 6.54	0.16
SD of retinal sensitivity	6.07 ± 3.28	6.66 ± 3.34	0.005

ACG			
Mean sensitivity (dB)	18.12 ± 7.51	17.28 ± 7.87	0.154
SD of retinal sensitivity	6.36 ± 3.14	7.35 ± 3.46	0.024

OAG: open-angle glaucoma; ACG: angle-closure glaucoma; SD: standard deviation; VF: visual field.

**Table 3:** Comparisons of ophthalmic measurements between two groups, before and after cataract surgery.

	OAG (*n* = 51)		ACG (*n* = 26)		*P* values for testing		
	Pre-op	Post-op	Pre-op	Post-op	Group difference	Pre-op and post-op difference	Group-time interaction
BCVA							
LogMAR	0.57 ± 0.32	0.18 ± 0.34	0.76 ± 0.41	0.30 ± 0.29	0.008	<0.001	0.817
Snellen	0.33 ± 0.18	0.79 ± 0.26	0.24 ± 0.17	0.60 ± 0.30	0.002	<0.001	0.046
IOP (mmHg)	13.33 ± 3.37	12.26 ± 3.64	14.87 ± 3.81	11.12 ± 2.92	0.828	<0.001	<0.001
No. of medications	1.31 ± 0.74	1.22 ± 0.61	1.62 ± 0.98	0.81 ± 0.63	0.723	<0.001	<0.001
MS slope (dB/year)	−0.39 ± 0.94	−0.47 ± 0.72	−1.39 ± 1.31	−0.34 ± 0.64	0.007	0.002	<0.001

OAG: open-angle glaucoma; ACG: angle-closure glaucoma; BCVA: best-corrected visual acuity; logMAR: logarithm of the minimum angle of resolution; IOP: intraocular pressure; MS: mean sensitivity.

## Data Availability

The data used to support the findings of this study are included within the article.

## References

[1] McCarty C. A., Mukesh B. N., Fu C. L., Taylor H. R. (1999). The epidemiology of cataract in Australia. *American Journal of Ophthalmology*.

[2] Mitchell P., Smith W., Attebo K., Healey P. R. (1996). Prevalence of open-angle glaucoma in Australia. *Ophthalmology*.

[3] Heijl A., Leske M. C., Bengtsson B. (2002). Reduction of intraocular pressure and glaucoma progression. *Archives of Ophthalmology*.

[4] Investigators A. (2001). The advanced glaucoma intervention study, 8: risk of cataract formation after trabeculectomy. *Archives of Ophthalmology*.

[5] Chen P. P., Budenz D. L. (1998). The effects of cataract extraction on the visual field of eyes with chronic open-angle glaucoma. *American Journal of Ophthalmology*.

[6] Hayashi K., Hayashi H., Nakao F., Hayashi F. (2001). Influence of cataract surgery on automated perimetry in patients with glaucoma. *American Journal of Ophthalmology*.

[7] Musch D. C., Gillespie B. W., Niziol L. M. (2006). Cataract extraction in the collaborative initial glaucoma treatment study. *Archives of Ophthalmology*.

[8] Koucheki B., Nouri-Mahdavi K., Patel G., Gaasterland D., Caprioli J. (2004). Visual field changes after cataract extraction: the AGIS experience. *American Journal of Ophthalmology*.

[9] Siddiqui M. A. R., Azuara-Blanco A., Neville S. (2005). Effect of cataract extraction on frequency doubling technology perimetry in patients with glaucoma. *British Journal of Ophthalmology*.

[10] Rehman Siddiqui M. A., Khairy H. A., Azuara-Blanco A. (2007). Effect of cataract extraction on SITA perimetry in patients with glaucoma. *Journal of Glaucoma*.

[11] Ang G. S., Shunmugam M., Azuara-Blanco A. (2010). Effect of cataract extraction on the glaucoma progression index (GPI) in glaucoma patients. *Journal of Glaucoma*.

[12] Rao H. L., Jonnadula G. B., Addepalli U. K., Senthil S., Garudadri C. S. (2013). Effect of cataract extraction on visual field index in glaucoma. *Journal of Glaucoma*.

[13] Kim J. H., Rabiolo A., Morales E. (2019). Cataract surgery and rate of visual field progression in primary open-angle glaucoma. *American Journal of Ophthalmology*.

[14] Lee J.-W., Morales E., Yu F. (2014). Effect of cataract extraction on the visual field decay rate in patients with glaucoma. *JAMA Ophthalmology*.

[15] Azuara-Blanco A., Burr J., Ramsay C. (2016). Effectiveness of early lens extraction for the treatment of primary angle-closure glaucoma (EAGLE): a randomised controlled trial. *The Lancet*.

[16] Nordmann J.-P., Mesbah M., Berdeaux G. (2005). Scoring of visual field measured through Humphrey perimetry: principal component varimax rotation followed by validated cluster analysis. *Investigative Opthalmology & Visual Science*.

[17] Stewart W. C., Rogers G. M., Crinkley C. M., Carlson A. N. (1995). Effect of cataract extraction on automated fields in chronic open-angle glaucoma. *Archives of Ophthalmology*.

[18] Carrillo M. M., Artes P. H., Nicolela M. T., LeBlanc R. P., Chauhan B. C. (2005). Effect of cataract extraction on the visual fields of patients with glaucoma. *Archives of Ophthalmology*.

[19] Smith S. D., Katz J., Quigley H. A. (1997). Effect of cataract extraction on the results of automated perimetry in glaucoma. *Archives of Ophthalmology*.

[20] Jaffe G. J., Alvarado J. A., Juster R. P. (1986). Age-related changes of the normal visual field. *Archives of Ophthalmology*.

[21] Collin H. B., Han C., Khor P. C. (1988). Age changes in the visual field using the Humphrey visual field analyser. *Clinical and Experimental Optometry*.

[22] Spry P. G. D., Johnson a. C. A. (2001). Senescent changes of the normal visual field: an age-old problem. *Optometry and Vision Science*.

[23] De Moraes C. G., Liebmann J. M., Liebmann C. A., Susanna R., Tello C., Ritch R. (2013). Visual field progression outcomes in glaucoma subtypes. *Acta Ophthalmologica*.

[24] Ballae Ganeshrao S., Senthil S., Choudhari N., Sri Durgam S., Garudadri C. S. (2019). Comparison of visual field progression rates among the high tension glaucoma, primary angle closure glaucoma, and normal tension glaucoma. *Investigative Opthalmology & Visual Science*.

[25] Saw S.-M., Gazzard G., Friedman D. S. (2003). Interventions for angle-closure glaucoma. *Ophthalmology*.

[26] Radhakrishnan S., Chen P. P., Junk A. K., Nouri-Mahdavi K., Chen T. C. (2018). Laser peripheral iridotomy in primary angle closure. *Ophthalmology*.

[27] Hayashi K., Hayashi H., Nakao F., Hayashi F. (2000). Changes in anterior chamber angle width and depth after intraocular lens implantation in eyes with glaucoma. *Ophthalmology*.

